# Calculating free energies of organic molecules on insulating substrates

**DOI:** 10.3762/bjnano.8.71

**Published:** 2017-03-21

**Authors:** Julian Gaberle, David Z Gao, Alexander L Shluger

**Affiliations:** 1 Department of Physics and Astronomy, University College London, Gower Street, London WC1E 6BT, United Kingdom

**Keywords:** entropy, free energy, molecular dynamics, organic molecules, potential of mean force, thermodynamic integration surface step

## Abstract

The challenges and limitations in calculating free energies and entropies of adsorption and interaction of organic molecules on an insulating substrate are discussed. The adhesion of 1,3,5-tri(4'-cyano-[1,1'-biphenyl]-4-yl)benzene (TCB) and 1,4-bis(4-cyanophenyl)-2,5-bis(decyloxy)benzene (CDB) molecules to step edges on the KCl(001) surface and the formation of molecular dimers were studied using classical molecular dynamics. Both molecules contain the same anchoring groups and benzene ring structures, yet differ in their flexibility. Therefore, the entropic contributions to their free energy differ, which affects surface processes. Using potential of mean force and thermodynamic integration techniques, free energy profiles and entropy changes were calculated for step adhesion and dimer formation of these molecules. However, converging these calculations is nontrivial and comes at large computational cost. We illustrate the difficulties as well as the possibilities of applying these methods towards understanding dynamic processes of organic molecules on insulating substrates.

## Introduction

In recent years molecular films and self-assembled monolayers have attracted a lot of attention due to their versatility, functionality, and technological potential. Understanding the behaviour of self-assembling molecules is important for catalysis [1], coatings [2], sensors [3–4] and molecular electronics [5–7]. To design and fabricate surface structures relevant for these technologies, a thorough understanding of the competing interactions at the surface is vital. Scanning tunnelling microscopy (STM) has been pivotal in achieving a high level of control over the molecular film structures on metal surfaces [8–14]. However, many applications require the use of insulating substrates where atomic force microscopy (AFM) provides vital information on film structure and growth modes.

Non-contact (NC)-AFM has provided rich information on the adsorption, self-assembly and film structure of various organic molecules on insulators [15–21]. The current status of NC-AFM studies of self-assembled films on insulating surfaces has recently been reviewed in [22]. However, theoretical modelling of the film growth processes still proves challenging. Most experiments are performed at room temperature, where entropic contributions can be significant [23–24]. Previous theoretical studies focussed on modelling adsorption [21,25–26], diffusion [27–28] and simple processes such as the flipping of a molecule [29]. The probability assigned to each of these processes is governed by the change in free energy Δ*G*, which can be derived from statistical mechanics [30]. In self-assembly processes, the right balance between molecule–molecule (MM) and molecule–surface (MS) interactions is critical to achieve large domains of ordered films. However, forming a molecular complex or a 2D film structure from freely rotating and translating molecules results in a loss in entropy as degrees of freedom within the molecules become constrained. This means that free energies can vary significantly from calculated enthalpy values, which may have a direct impact on our understanding of the balance of interactions that govern self-assembly.

Methods to compute the free energy from molecular dynamics (MD) simulations have been developed for many years and the most popular ones are summarised in [31]. Instead of calculating free energies directly, they can be expressed as averages over ensembles of atomic configurations. Such ensembles can be obtained from Monte Carlo (MC) or MD simulations. Despite this seemingly simple process, calculating free energies is far from trivial. In order to obtain converged results the ergodicity needs to be satisfied. The ergodic principle states that an infinite trajectory (in time) should sample all possible states of a system. However, in practice trajectories are finite and it is difficult to determine how long such simulations need to be.

Attempts to compute free energies have been made for, i.e., proteins [32–34], ion solvation [35–36], small molecular clusters [37–38] and small molecules on surfaces [39–41]. While well-converged results can be obtained for small systems, the larger the configurational space the more challenging the calculations become and convergence is not guaranteed [32,42]. In particular, when the free-energy landscape varies by several *kT* along the reaction coordinate, MD is often insufficient for sampling the high-energy states. The challenge of achieving convergence can be addressed by running constrained MD, where the system evolves along a defined reaction coordinate in order to improve the sampling of high-energy, low- probability states [31].

In this paper we investigated the free energy of of 1,3,5-tri(4'-cyano-[1,1'-biphenyl]-4-yl)benzene (TCB) and 1,4-bis(cyanophenyl)-2,5-bis(decyloxy)benzene (CDB) molecules on an insulating KCl(001) surface and partitioned it into entropy and enthalpy contributions. Previously we found that entropy loss can give significant contributions to free energy of adsorption of these molecules at high temperatures [43]. Here we investigate free-energy profiles for the adhesion of these molecules to a monatomic step edge and the formation of dimers on a clean terrace. These processes are important during the early stages of self-assembly and a better understanding of their energetics at nonzero temperatures will help to elucidate the mechanisms responsible for these processes. Thermodynamic integration and potential of mean force (PMF) calculations were employed in order to calculate entropies and free energies from classical MD. The dependence of the accuracy of these quantities on simulation time is investigated in order to check for convergence. The results show that subtle features in the free-energy landscape can be resolved with very long trajectories even if the calculations are not fully converged. Furthermore, entropic contributions to free energy can significantly lower the adhesion energy of molecules at step edges and cannot be ignored when considering film growth at step edges and terraces.

## Methods

### Classical force fields

In order to calculate free energies of molecular processes on surfaces, long-timescale MD simulations are needed. Therefore classical force fields are used since the computational cost associated with ab initio methods is too high. The LAMMPS code was used for all calculations [44] along with a combination of several classical force fields. A Buckingham potential was used to describe the interactions inside the KCl slab, as parameterised by Catlow and co-workers [45]. The inter- and intramolecular interactions of CDB and TCB molecules were described using the CHARMM force field [46]. Since there was no force field available for the interactions of organic molecules with KCl, we parameterised Morse interatomic potentials for each atom type inside molecules and the KCl surface using a genetic algorithm method. A detailed discussion of this potential-fitting method can be found in prior publications [25,43].

Briefly, a fitting dataset composed of 240 configurations was generated using density functional theory (DFT). These calculations were performed using the CP2K code [47], the PBE GGA density functional [48], the MOLOPT basis set [49], and semi-empirical long-range dispersion corrections [50]. Since vdW interactions are poorly described in DFT, we assessed the accuracy of the semi-empirical corrections against higher-accuracy second-order Møller–Plesset perturbation theory (MP2) [51–52] calculations of smaller molecular fragments [43]. A genetic algorithm was employed to fit parameters against that dataset, where the fitness criterion was defined as the force between the molecule and the surface. The total population size was 1024 elements and they were evolved for 1000 generations. In order to avoid over-fitting of the potential, high-energy configurations obtained from ab initio DFT were included in the dataset. In each generation 5% of the population was randomly mutated in order to reduce artificial convergence. The atomic charges of atoms within the TCB and CDB molecules were assigned using Mulliken population analysis of the DFT dataset and classical charges of ±1 were used for KCl. The adsorption geometries obtained using this force field reproduced the results of vdW corrected DFT calculations well and adsorption energies matched within 7%.

All calculations employed a four-layer slab of KCl, where the bottom (001) surface was frozen to avoid the system from drifting in the simulation box. MD simulations were run with a time step of 1 fs, and NVT thermostats were applied to the surface and the molecules separately.

### Thermodynamic integration and PMF

Previously, we have shown that entropy loss upon adsorption of CDB and TCB molecules on a clean terrace greatly contributes to the adsorption free energy and that the magnitude of this energy loss is comparable to the enthalpy at high temperatures [43].

Smith et al. [38] proposed an accurate method to compute entropy changes via thermodynamic integration as given by:

[1]
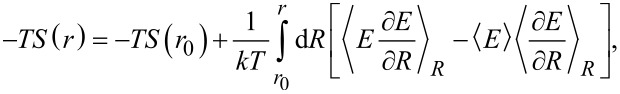


where *E* is the potential energy and *R* is a defined reaction coordinate. In this work, the reaction coordinate was defined as the centre of mass (COM) separation between two molecules or the distance between a step edge and the COM of a molecule. Averages were taken from 50 ns MD simulations at each separation *R*. The MD simulation time was then increased to 80 ns and 100 ns in order to check for convergence. Zero entropy was defined to be at a configuration where the force along the reaction coordinate is zero (i.e., at large separations). The reaction coordinate *R* was varied by 0.1Å increments and the COM was fixed at each coordinate *R*.

Potential of mean force (PMF) calculations [53–54] were then performed in order to investigate the free-energy changes of dynamic processes of TCB and CDB molecules adsorbed on the KCl(100) surface. Again the system is forced to move along a defined reaction path *R*, where the COM of the molecule is constrained by the condition *R*(**r**) = *R*_c_. The free-energy difference between two states along the reaction coordinate *R* is defined as

[2]
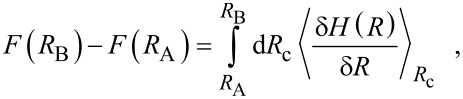


where 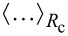 is an ensemble average over the constrained simulation corresponding to a parameter value *R*_c_ [55]. The integrand is the constraining force required to satisfy the condition *R*(**r**) = *R*_c_ and can be directly obtained from MD simulations.

## Results and Discussion

### Adhesion to step edges for TCB and CDB

Step edges and kink sites play an important role in the dynamic processes of large organic molecules on insulating surfaces. At these sites the molecules can interact with the atoms of the step layer as well as terrace layer and become more strongly bound [56]. Furthermore, they can exhibit one-dimensional motion when diffusing along step edges, become trapped [57–58], or even reconstruct the step geometry [59]. In order to model these dynamic processes one needs to move from static methods to molecular dynamics in order to obtain a better understanding of the free-energy landscape.

**Figure 1:**
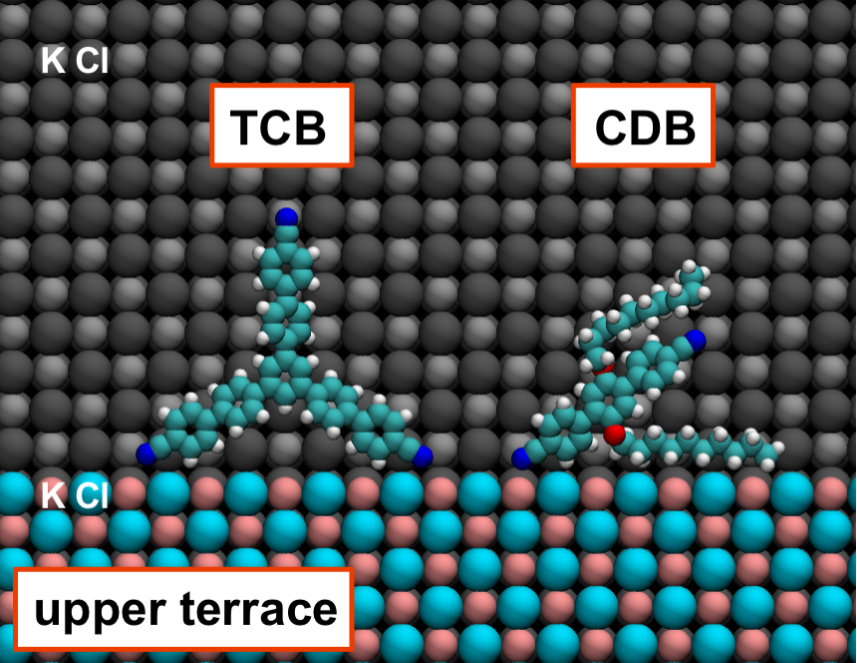
Lowest-energy adsorption geometries of a CDB and a TCB molecule at a monatomic KCl step edge. Colour code: Emerald = carbon, red = oxygen, blue = nitrogen, white = hydrogen, silver/coral = potassium, grey/cyan = chlorine. The normal of the surface is perpendicular to the screen.

We modelled the interaction of TCB and CDB molecules with step edges and investigated the enthalpic and entropic contributions to the adsorption free energy at room temperature. The adsorption geometries of CDB and TCB molecules at a monatomic KCl step edge are shown in Figure 1. The flexibility of the hydrocarbon chains of CDB allows the molecule to structurally adapt and interact strongly with the KCl step edge resulting in an adsorption enthalpy of 4.0 eV compared to 3.1 eV for an isolated molecule on a clean KCl(001) terrace. However, this adsorption enthalpy is offset by a decrease in entropy since one of the hydrocarbon chains becomes constrained by the step edge. Therefore a more detailed investigation of the balance of enthalpic and entropic contributions to the adsorption free energy is required.

The adsorption enthalpy of TCB also increases from 4.5 eV on a clean terrace to 4.8 eV at a step edge when two legs of the TCB molecule adhere to cation sites of the step (Figure 1). The increase in adsorption enthalpy is smaller for rigid TCB because it cannot adapt the geometry to maximise interactions with the step edge. This shows that for the more rigid TCB the entropic contribution to step adhesion should be lower than that for CDB.

**Figure 2:**
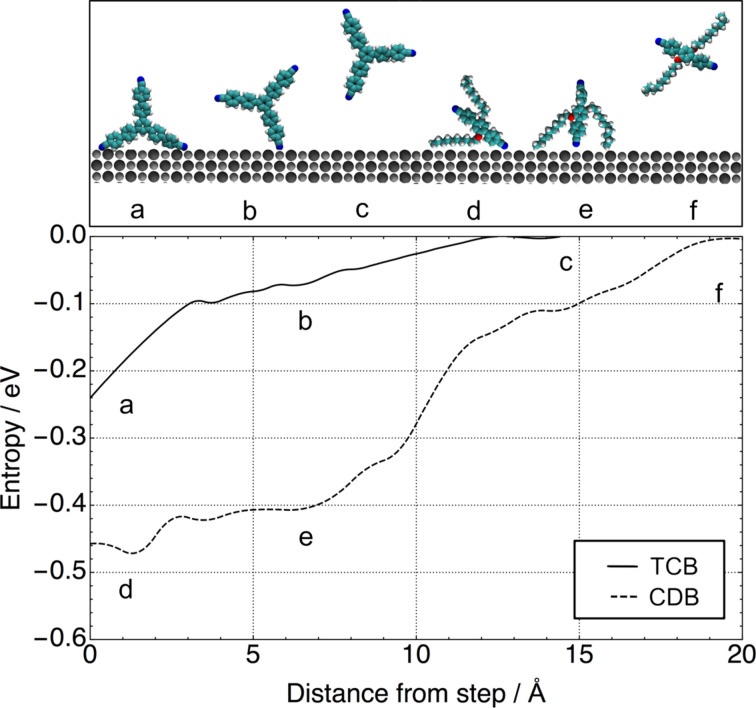
Change in entropy for step adhesion at 300 K for CDB (dotted line) and TCB (solid line) as a function of molecule–step separation. Zero on the *x*-axis was chosen as the separation of the molecule to the step edge in its minimum-energy adsorption geometry and zero entropy was defined as the separation, where the force between the step and the molecule is zero.

In order to investigate the entropic contributions to step adhesion, thermodynamic integration methods were used to calculate the change in entropy as a function of molecule–step separation (Figure 2). As a CDB molecule approaches the step edge one hydrocarbon arm starts to interact with it, leading to an initial drop in entropy of about 0.4 eV. Subsequently the attraction between a cyano group and a cation site reduces the entropy further by about 0.05 eV yielding a total decrease in entropy of around 0.45 eV. As expected the largest contribution to the change in entropy can be assigned to constraining one hydrocarbon chain at the step edge. On a clean terrace the hydrocarbon chains are able to move rapidly in an erratic motion around the central body of the molecule, accessing many different conformations. As the molecule becomes trapped at the step edge, the hydrocarbon chain cannot access as many conformations and thus its entropy is reduced.

In the case of TCB, the overall change in entropy is much smaller (Figure 2). As one leg of the molecule attaches to a cation site at a step edge, its entropy is reduced by about 0.1 eV and as the second leg adheres to the step edge it is reduced by another 0.14 eV. The observed change in entropy is much smoother than with CDB because of the fact that the rigidity of TCB does not allow the molecule to adopt many different conformations. TCB will be less affected by the step edge and displays a smaller overall change in entropy.

Comparing the calculated values for change in entropy to step adhesion enthalpy, one can note that the entropy reduction due to constraining the hydrocarbon chains significantly offsets the increase in adsorption enthalpy. While the CDB molecule loses about 0.55 eV of entropy, it gains 0.9 eV from adsorbing at the step edge. Thus the molecule is expected to get trapped by step edges. In contrast, the entropic and enthalpic contributions to step adhesion for TCB are nearly the same. One would expect an equilibrium situation, where molecules both adhere to the step edge and move around freely on the terrace. This illustrates that using static methods, such as DFT and energy minimisation, can be insufficient when investigating dynamic processes, such as step adhesion. Changes in entropic and enthalpic contributions to adsorption free energy upon step adhesion can be small and their relative value determines whether one state is more favourable than the other.

However, the convergence of thermodynamic integration calculations must be checked thoroughly. When increasing the MD simulation time at each step along the reaction coordinate from 50 ns to 80 ns the calculated values for change in entropy differed only marginally. This gives us confidence that convergence has been reached and that longer trajectories will not give much improvement in accuracy.

### Free-energy calculations

Instead of calculating the enthalpic and entropic contributions separately, one can also attempt to explore the free-energy landscape of these molecular processes directly. However, calculating free energies from molecular dynamics is still not straightforward. In order to obtain fully converged energies, all possible conformations and velocities need to be sampled. This can be particularly difficult when the free-energy difference between the initial state A and the final state B is large or the system has to transverse over large barriers. In these cases the system may get stuck in a deep potential well and higher energy states will not be sufficiently sampled. For this investigation, a constrained MD was applied in order to improve the sampling of the potential energy surface at each separation *R*.

Knowing the potential of mean force obtained using constrained MD simulations (along the reaction coordinate *R*) one can calculate the free energy of the simulated transition as:

[3]
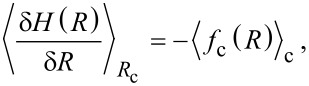


where 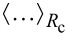 denotes an ensemble average over the constrained simulation and *f*_c_(*R*) is the constraining force at a given *R*.

**Figure 3:**
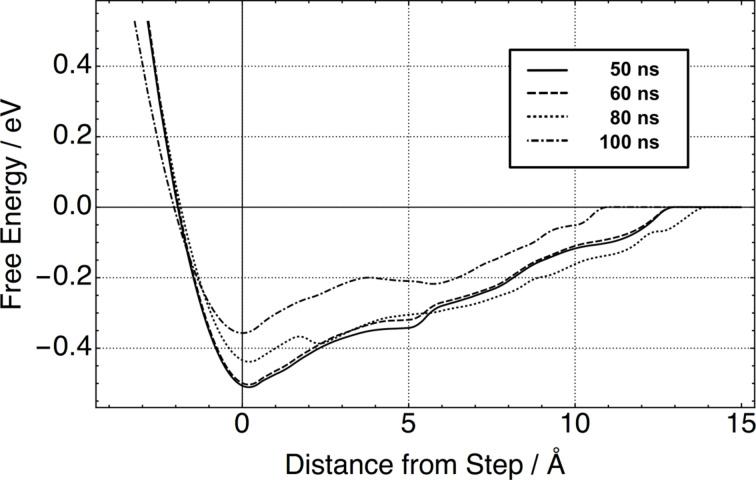
Change in free energy for a single TCB molecule adhering to a step edge at 300 K. Zero on the x-axis was chosen as the separation of the molecule to the step edge in its minimum-energy adsorption geometry.

The PMF scheme was applied to calculate the free energy of step adhesion for TCB at 300 K using 50 ns to 100 ns long MD simulations with the results shown in Figure 3. The distance between the COM of the molecule and the step edge was chosen as the reaction coordinate *R* and the zero on the *x*-axis corresponds to this distance in the minimum energy configuration. The molecule was not constrained in any other directions, allowing it to fully explore the energy landscape.

The first thing to notice is the spread of the obtained free-energy curves. Increasing the MD trajectory from 50 ns to 100 ns leads to a drop in the free energy of step adhesion from 0.5 eV to 0.35 eV. As MD simulation times are increased, the free energy continues to decrease as more and more of the higher-energy, low-probability configurational space is sampled. This indicates that performing short MD simulations leads to an overestimation of the free energy. Even after 100 ns of MD, the free energy is still overestimated as it is still larger than the enthalpic contribution, which was calculated to be 0.25 eV. Thus full convergence could not be achieved.

The free energy profile for the 100 ns MD case displays a double-dip feature corresponding to two stable TCB adsorption geometries with one leg (metastable) and two legs attached to step-edge cation sites. A small barrier of 0.03 eV needs to be traversed to go from having one leg attached to two, which is within the thermally accessible energy range. The existence of this second minimum was confirmed using energy-minimisation calculations. The molecule will adsorb with one leg on the step edge with an adsorption energy of 4.7 eV. This illustrates that subtle features in the free-energy profile can be resolved using very long MD simulations even if the absolute free-energy value itself is not fully converged.

**Figure 4:**
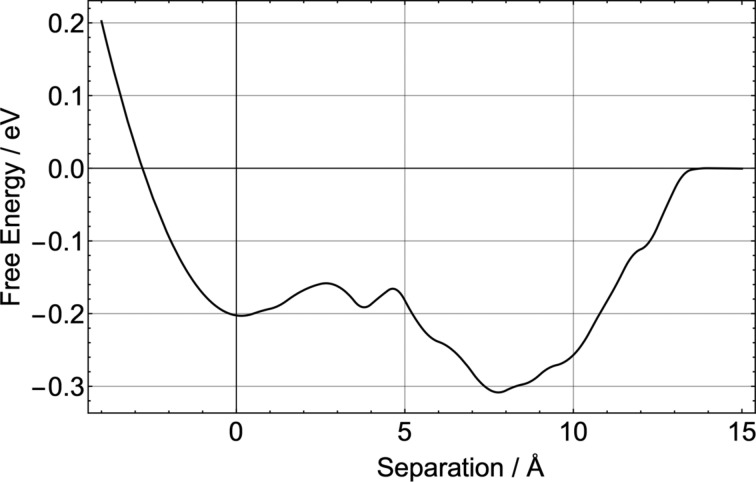
Change in free energy for a single CDB molecule adhering to a step edge at 300 K. Zero on the *x*-axis was chosen as the separation of the molecule to the step edge in its minimum-enthalpy adsorption geometry.

Similarly the free energy of step adhesion was investigated for the more flexible CDB molecule. With two very mobile hydrocarbon arms, CDB has more degrees of freedom than TCB. In Figure 4 the change in free energy is plotted as a function of the molecule–step distance as obtained from a 100 ns MD trajectory. As one arm of the molecule attaches to the step edge, a large drop in entropy was observed. However, the free-energy profile seems to suggest a much more favourable interaction. In fact it appeared even more stable than the geometry found using energy-minimisation calculations. In principle it could be possible that entropy shifts the balance of interactions in favour of a different adsorption geometry. However, in this case we believe that this can be attributed to poor sampling of the phase space when one arm gets attached to the step edge. It can be seen in the MD trajectories that as soon as the arm attaches to the step edge the molecule becomes trapped in that potential minimum and sampling of other configurations is poor, resulting in an overestimation of the free energy. Therefore, fully exploring the phase space for complex flexible molecules would require further increasing the MD trajectory time or employing a different method, such as umbrella sampling.

### Converging entropy calculations

**Figure 5:**
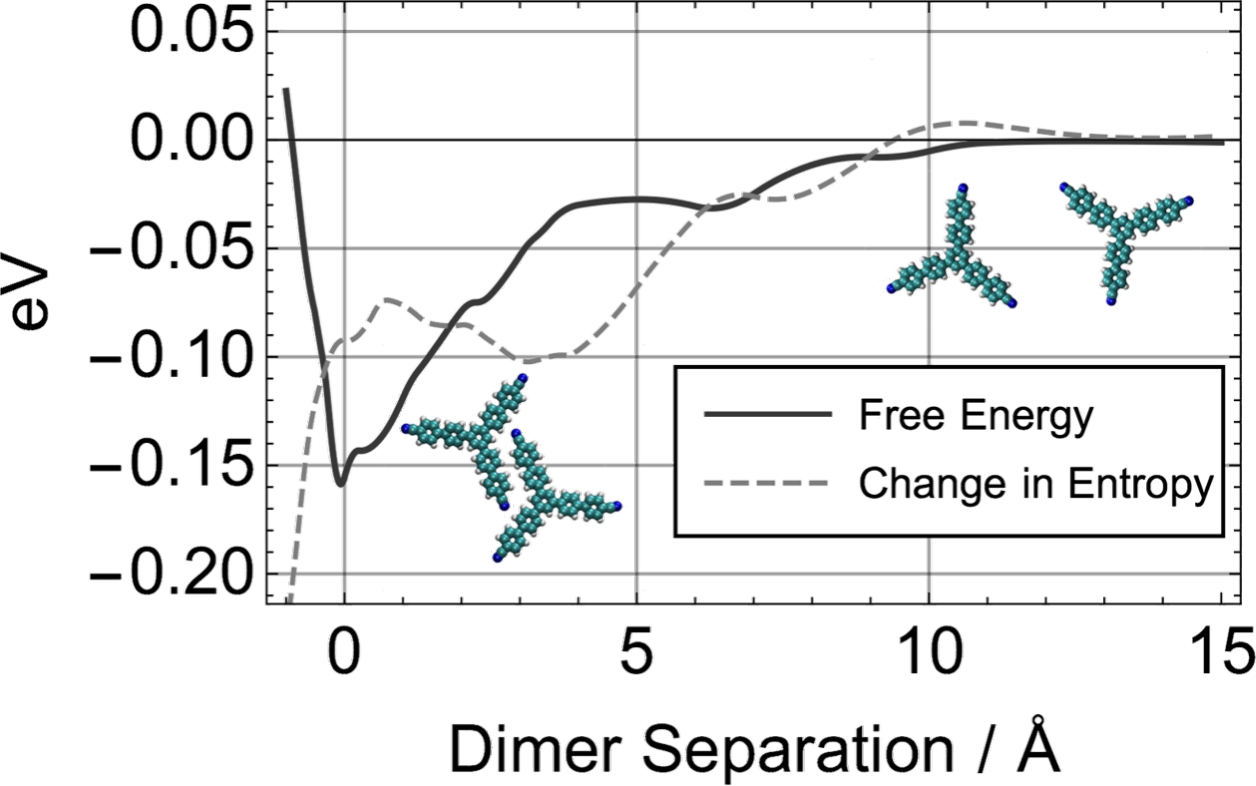
Change of free energy and entropy of TCB dimer formation as a function of molecular separation at 300 K, where zero on the *x*-axis was chosen as the lowest-energy configuration. The free energy and entropy curves were aligned to be zero at large molecular separation, where the force between the molecules approaches zero.

In order to illuminate the problem of obtaining converged free energies from molecular dynamics simulations, another setup was tested: TCB molecules will readily form dimers on terraces, in which two molecules will align next to each other (see inset of Figure 5). This process occurs spontaneously in MD simulations at 300 K when two TCB molecules are placed near each other on a KCl terrace. The dimer represents an energetically favourable state with a relatively small barrier of formation.

**Figure 6:**
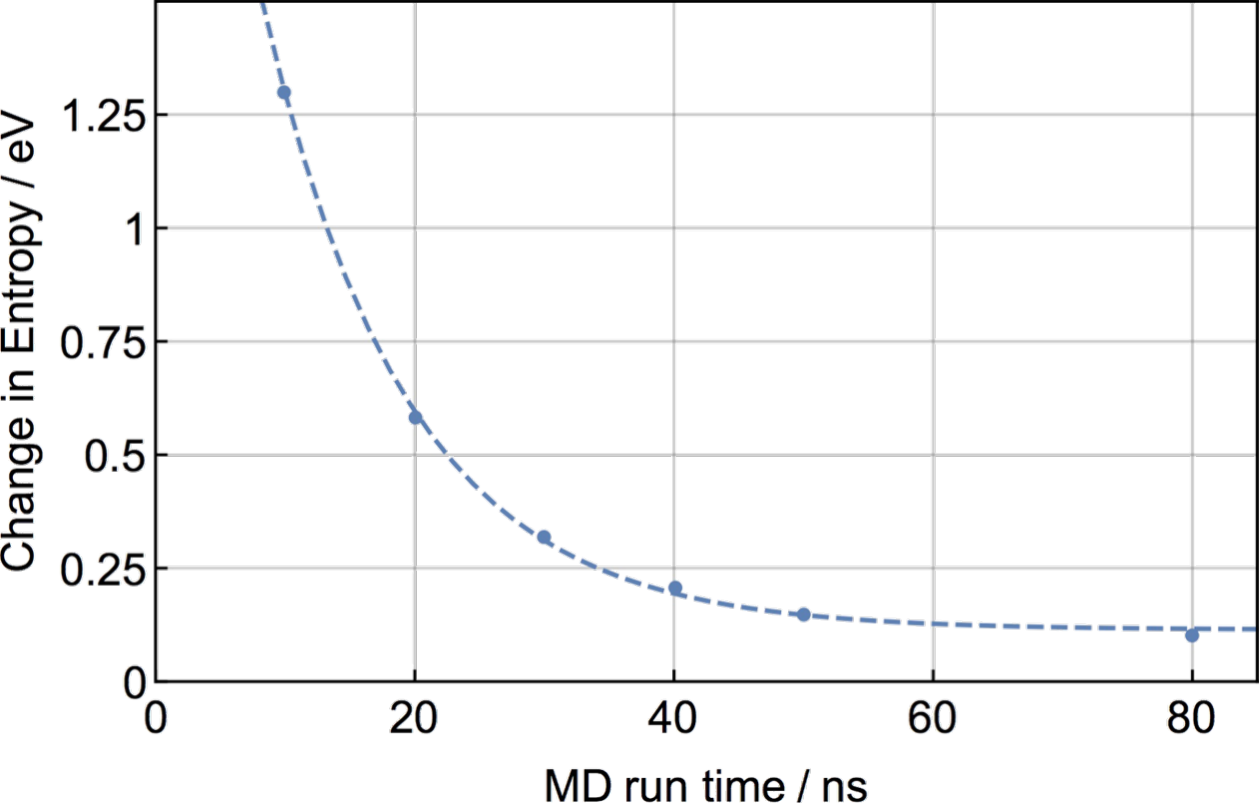
Convergence of entropy change upon dimer formation as a function of the MD simulation time at 300 K. The dashed line is an exponential fit to the data and shown for guidance only.

The free energy of dimer formation was calculated from 80 ns MD simulations along with the entropic and enthalpic contributions (Figure 5). The enthalpy of dimer binding amounts to 0.25 eV, while at room temperature the entropic contribution to the free energy was calculated to be −0.1 eV, which gives a total free energy of binding of 0.15 eV at 300 K. Indeed the same value was obtained from directly calculating the free energy using PMF simulations. The reaction coordinate was the intermolecular separation and the molecules were free to translate and rotate on the surface. Plotting the change in entropy as a function of MD run time gives an indicator of whether convergence was reached, as illustrated in Figure 6. It is important to note that the exponential decay curve was fitted for guidance only and may not be a mathematically accurate representation of the decay rate. At less than 10 ns the change in entropy can be overestimated by an order of magnitude. Only for very long run times (*<*50 ns) is convergence slowly reached.

## Conclusion

Thermodynamic integration and potential of mean force calculations have been employed to study the adsorption of large organic molecules at monatomic step edges as well as the dimer formation process. Despite recent progress in computing free energies and an increase in computer power, it is still not trivial to calculate free energies of processes for large organic molecules adsorbed on an insulating surface. Convergence can be slow and in some cases impossible to obtain, as was found for the case of CDB.

Nevertheless it is vital to gain better understanding of the free-energy landscape and the competing interactions at higher temperatures. Many experiments are performed on systems at room temperature, where thermal motion and, consequently, entropy cannot be ignored. The balance of molecule–surface and molecule–molecule interactions determines the dynamics and ultimately the growth process for molecular films. This balance can shift as one moves from cryogenic temperatures and frozen molecules to NC-AFM observations at room temperature.

In this work we showed that TCB molecules have a favourable step-adsorption enthalpy, which is compensated at room temperature by a nearly equal loss in entropy during step adhesion, resulting in a negligible step-adhesion free energy. These molecules were also found to assemble in dimers on a clean terrace, where the free-energy profile shows a more favourable interaction. Static calculations, on the other hand, led to the conclusion that TCB molecules are more stable at step edges, since the step-adhesion enthalpy is larger than the dimer-formation enthalpy. These results highlight the importance of accurate predictions of entropy changes and free energy in modelling the early stages of self-assembly and in determining film morphologies. In many cases the interaction between molecules and between molecules and surface features are weak and entropy changes are comparable to enthalpy gains resulting in variety of structures and their strong dependence on temperature.

## References

[1] Besenbacher F, Lauritsen J V, Wendt S (2007). Nano Today.

[2] Maboudian R, Ashurst W R, Carraro C (2000). Sens Actuators, A.

[3] Mason A, Mukhopadhyay S C, Jayasundera K P (2015). Sensing Technology: Current Status and Future Trends I.

[4] Chaki N K, Vijayamohanan K (2002). Biosens Bioelectron.

[5] Joachim C, Gimezewski J K, Aviram A (2000). Nature.

[6] Heath J R (2009). Annu Rev Mater Sci.

[7] Song H, Reed M A, Lee T (2011). Adv Mater.

[8] de Wild M, Berner S, Suzuki H, Yanagi H, Schlettwein D, Ivan S, Baratoff A, Guntherodt H-J, Jung T A (2002). ChemPhysChem.

[9] Bobisch C, Wagner T, Bannani A, Möller R (2003). J Chem Phys.

[10] Grill L, Dyer M, Lafferentz L, Persson M, Peters M V, Hecht S (2007). Nat Nanotechnol.

[11] Abel M, Clair S, Ourdjini O, Mossoyan M, Porte L (2011). J Am Chem Soc.

[12] Ourdjini O, Pawlak R, Abel M, Clair S, Chen L, Bergeon N, Sassi M, Oison V, Debierre J M, Coratger R (2011). Phys Rev B.

[13] Payamyar P, Kaja K, Ruiz-Vargas C, Stemmer A, Murray D J, Johnson C J, King B T, Schiffmann F, VandeVondele J, Renn A (2014). Adv Mater.

[14] Zheng Z, Opilik L, Schiffmann F, Liu W, Bergamini G, Ceroni P, Lee L-T, Schütz A, Sakamoto J, Zenobi R (2014). J Am Chem Soc.

[15] Hinault A, Pujol A, Chaumeton F, Martrou D, Gourdon A, Gauthier S (2012). Beilstein J Nanotechnol.

[16] Hauke C M, Bechstein R, Kittelmann M, Storz C, Kilbinger A F M, Rahe P, Kühnle A (2013). ACS Nano.

[17] Pawlak R, Nony L, Bocquet F, Oison V, Sassi M, Debierre J-M, Loppacher C, Porte L (2010). J Phys Chem C.

[18] Rahe P, Nimmrich M, Kühnle A (2012). Small.

[19] Loppacher C, Zerweck U, Eng L M, Gemming S, Seifert G, Olbrich C, Morawetz K, Schreiber M (2006). Nanotechnology.

[20] Hentschke R, Schürmann B L, Rabe J P (1992). J Chem Phys.

[21] Amrous A, Bocquet F, Nony L, Para F, Loppacher C, Lamare S, Palmino F, Cherioux F, Gao D Z, Canova F F (2014). Adv Mater Interfaces.

[22] Rahe P, Kittelmann M, Neff J L, Nimmrich M, Reichling M, Maass P, Kühnle A (2013). Adv Mater.

[23] Roos M, Breitruck A, Hoster H E, Behm R J (2010). Phys Chem Chem Phys.

[24] Campbell C T, Sellers J R V (2012). J Am Chem Soc.

[25] Gao D Z, Federici Canova F, Watkins M B, Shluger A L (2015). J Comput Chem.

[26] Barth C, Gingras M, Foster A S, Gulans A, Félix G, Hynninen T, Peresutti R, Henry C R (2012). Adv Mater.

[27] Otero R, Hümmelink F, Sato F, Legoas S B, Thostrup P, Laegsgaard E, Stensgaard I, Galvão D S, Besenbacher F (2004). Nat Mater.

[28] Ma M, Tocci G, Michaelides A, Aeppli G (2016). Nat Mater.

[29] Abbasi-Pérez D, Manuel Recio J, Kantorovich L (2015). Phys Chem Chem Phys.

[30] Van Gunsteren W F, Daura X, Mark A E (2002). Helv Chim Acta.

[31] Trzesniak D, Kunz A-P E, van Gunsteren W F (2007). ChemPhysChem.

[32] Genheden S, Ryde U (2012). Phys Chem Chem Phys.

[33] Dinner A R, Šalib A, Smitha L J, Dobsona C M, Karplus M (2000). Trends Biochem Sci.

[34] Boczko E M, Brooks C L (1995). Science.

[35] Guàrdia E, Rey R, Padró J A (1991). Chem Phys.

[36] Åqvist J (1990). J Phys Chem.

[37] Tang H Y, Ford I J (2015). Phys Rev E.

[38] Smith D E, Zhang L, Haymet A D J (1992). J Am Chem Soc.

[39] Alfè D, Gillan M J (2006). J Phys: Condens Matter.

[40] Jonsson H, Mills G, Nylén M (1994). Phys Rev Lett.

[41] Fox H, Gillan M J, Horsfield A P (2009). Surf Sci.

[42] Meirovitch H, Cheluvaraja S, White R P (2009). Curr Protein Pept Sci.

[43] Gaberle J, Gao D Z, Watkins M B, Shluger A L (2016). J Phys Chem C.

[44] Plimpton S (1995). J Comput Phys.

[45] Catlow C R A, Diller K M, Norgett M J (1977). J Phys C.

[46] Brooks B R, Bruccoleri R E, Olafson B D, States D J, Swaminathan S, Karplus M (1983). J Comput Chem.

[47] Lippert G, Hutter J, Parrinello M (1997). Mol Phys.

[48] Perdew J P, Burke K, Ernzerhof M (1996). Phys Rev Lett.

[49] VandeVondele J, Hutter J (2007). J Chem Phys.

[50] Grimme S (2006). J Comput Chem.

[51] Del Ben M, Hutter J, VandeVondele J (2012). J Chem Theory Comput.

[52] Del Ben M, Hutter J, VandeVondele J (2015). J Chem Phys.

[53] Doudou S, Burton N A, Henchman R H (2009). J Chem Theory Comput.

[54] Ben-Tal N, Honig B, Bagdassarian C K, Ben-Shaul A (2000). Biophys J.

[55] Roux B (1995). Comput Phys Commun.

[56] Barth J V, Costantini G, Kern K (2005). Nature.

[57] Hlawacek G, Puschnig P, Frank P, Winkler A, Ambrosch-Draxl C, Teichert C (2008). Science.

[58] Meyer zu Heringdorf F-J, Reuter M C, Tromp R M (2001). Nature.

[59] Trevethan T, Such B, Glatzel T, Kawai S, Shluger A L, Meyer E, de Mendoza P, Echavarren A M (2011). Small.

